# How Transmembrane Inner Ear (TMIE) plays role in the auditory system: A mystery to us

**DOI:** 10.1111/jcmm.16610

**Published:** 2021-05-13

**Authors:** Mohammad Farhadi, Ehsan Razmara, Maryam Balali, Yeganeh Hajabbas Farshchi, Masoumeh Falah

**Affiliations:** ^1^ ENT and Head and Neck Research Center and Department The Five Senses Health Institute Hazrat Rasoul Akram Hospital Iran University of Medical Sciences Tehran Iran; ^2^ Australian Regenerative Medicine Institute Monash University Clayton VIC Australia; ^3^ Department of Cellular and Molecular Biology Tehran Medical Sciences Islamic Azad University Tehran Iran

**Keywords:** DFNB6, hair cells, hearing impairment, mechanotransduction, nAChR, TMIE

## Abstract

Different cellular mechanisms contribute to the hearing sense, so it is obvious that any disruption in such processes leads to hearing impairment that greatly influences the global economy and quality of life of the patients and their relatives. In the past two decades, transmembrane inner ear (TMIE) protein has received a great deal of research interest because its impairments cause hereditary deafness in humans. This evolutionarily conserved membrane protein contributes to a fundamental complex that plays role in the maintenance and function of the sensory hair cells. Although the critical roles of the TMIE in mechanoelectrical transduction or hearing procedures have been discussed, there are little to no review papers summarizing the roles of the TMIE in the auditory system. In order to fill this gap, herein, we discuss the important roles of this protein in the auditory system including its role in mechanotransduction, olivocochlear synapse, morphology and different signalling pathways; we also review the genotype‐phenotype correlation that can per se show the possible roles of this protein in the auditory system.

## 1 INTRODUCTION

Hearing Impairment (HI) as the most common form of sensory disorder affects around one in 500 newborns.^1^, ^2^, ^3^ Presently, more than 5% of the world's population (~466 million people) suffer from the HI; according to the World Health Organization, this rate is expected to increase to more than 900 million by 2050.^4^ The effective therapeutic procedure for HI is still based on hearing amplification and cochlear implantation, though they cannot restore the natural hearing power.^5^, ^6^ Understanding the molecular mechanisms of the hearing process not only does shed light on the intracellular mechanisms but also makes it possible to manipulate them for HI treatment.^7^

More than half of cases of prelingual hearing loss are imputed to impaired genetic factors.^8^, ^9^ In the last few decades, high genetic heterogeneity of HI was introduced and more than 150 genes have been identified in association with HI.^10^ Eighty per cent of the prelingual HI cases are classified into non‐syndromic in which the HI is manifested as the only detectable symptom.^11^ Non‐syndromic hearing loss loci are determined by DFN (DeaFNess) and further arranged concerning the mode of inheritance (DFNA: autosomal dominant; DFNB: autosomal recessive; and DFNX: X‐linked); the following number indicates the order of gene mapping and/or discovery.^11^

**FIGURE 1:**
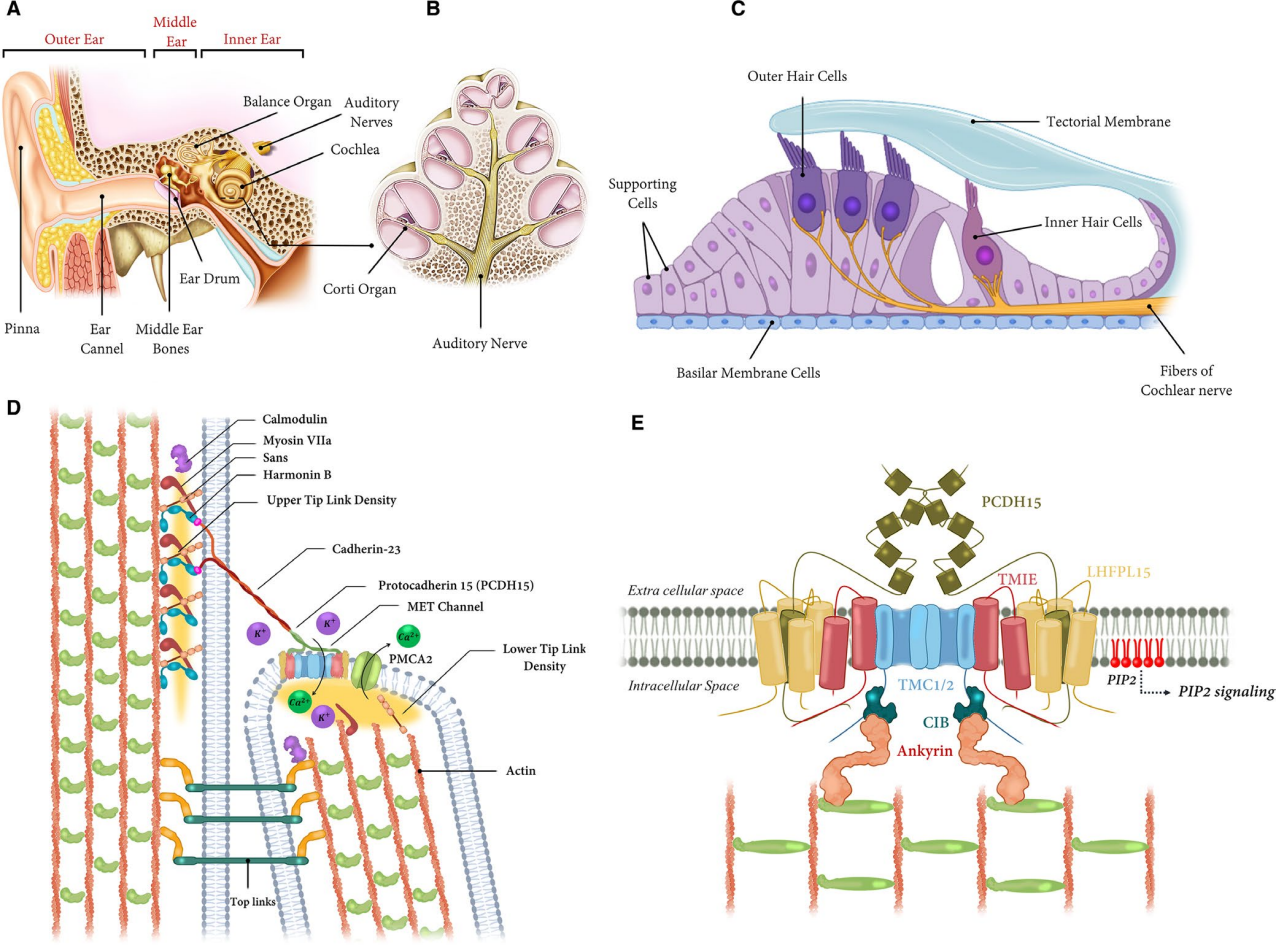
Schematic illustration of the inner ear, cochlear turn cross‐section, stereocilia and mechano‐electrical transduction (MET) channel. A, Anatomically, the ear consists of three distinct parts including the outer ear, middle ear, and inner ear. The inner ear has two main parts: the cochlea, which is the hearing portion, and the semicircular canals that are the balance portion. B, A cross‐section shows the 2.5 turns containing the cochlea duct. C, In the Corti organ, outer hair cells are arranged in three longitudinal rows, whereas the inner hair‐cells in a single one, extending along the whole cochlea. D, Schematic diagram of stereocilia of the hair cell. Cadherin 23 and protocadherin‐15 (PCDH15) comprise the tip link, which inserts into the stereocilia membrane at the sites of the upper and lower tip densities. Scaffolding proteins—including Myosin VIIa, Harmonin and Sans—bind to the cytoplasmic domain of Cadherin 23 and anchor the tip link. The ‘Upper’ and the ‘Lower Tip Link Density’ regions are shown by yellow highlighted zones. Calmodulin binds to the Ca^2+^ and also stereocilin protein that, in turn, attaches two stereocilia. E, A model of the MET complex of hair cells. TMC1/2 dimers (TMC1/2) can interact with PCDH15 dimers and TMIE. TMC1/2 dimers by tension exerted perpendicular to the membrane with extracellular tip‐link (PCDH15) tension and TMC1/2 tethered intracellularly to CIB2 (dark blue) and Ankyrin (orange) and the actin cytoskeleton

The cochlea within the inner ear contains the cells responsible for the perception of sound (Figure 1A, B). Hearing occurs when hair cells of the inner ear convert the sound‐induced vibrations into the nerve impulses that are conveyed to the brain for further interpretation. Hair cells are the mechanosensory cells in auditory and vestibular systems in the inner ears of all the vertebrates; they are also detectable in a functionally homologous way in the lateral line organ of the fish.^12^ The appellation of hair cells was due to the hair bundles that are present on its apical surface, which involves the stereocilia that are mechanically sensitive organelles of hair cells in the rows with a staircase‐like pattern (Figure 1C). Any damages to the hair cells cause hearing or balance disorder.^13^ The stereocilia are connected by the extracellular filament called the tip links. These structures recognize any surrounding movement through mechanotransduction, a transformation of the mechanical force into electrical signals; this process is essential for the sense of proprioception, hearing, touch and balance.^14^

One or two mechanotransduction channels are located in every stereocilium of hair cells.^15^ They are located on the surface of shorter stereocilia next to the lower end of tip links^15^ (Figure 1D). Tip links transmit the mechanical power onto the transduction channels that can be opened by the stereocilia deflection, and it therefore allows to enter the small positively charged ions like Ca^2+^ and K^+^ from the surrounding endolymph. Finally, the depolarized cells send the electrical output to the brain through the eighth cranial nerve (reviewed in Ref. [16]). Deflections of stereocilia to the longest one open the transduction channels, whereas deflections in the opposite direction close them.^14^

Numerous studies have been conducted to understand the mechanisms of the hair cell mechanotransduction; for example, the research on the genes associated with the inherited HI has introduced several components of the mechanotransduction machinery of hair cells. This complex consists of the Protocadherin‐15 (PCDH15), Cadherin 23 (CDH23), LHFP‐like protein 5 (LHFPL5), Transmembrane inner ear (TMIE), Transmembrane channel‐like 1 (TMC1), TMC2, Usher syndrome 1C (USH1C), Myosin VIIA (MYO7A), Usher syndrome 1G (USH1G) and Calcium and integrin‐binding protein 2 (CIB2) (Figure 1E). The transduction channel may contain additional components that have not been identified yet.

Transmembrane Inner Ear, an evolutionarily conserved protein, is one of the main components of the mechanotransduction complex of hair cells. Loss‐of‐function mutations in the *TMIE* cause autosomal recessive deafness‐6 (DFNB6; OMIM: 600971).^17^, ^18^, ^19^, ^20^ Many studies have been performed to understand the roles of TMIE in the maintenance, maturation and development of the inner ear sensory hair cells, but there is no review article summarizing these; to fill, the present review focuses on the genotype‐phenotype correlation, the critical role of the TMIE in sensory hair cells in the auditory process, and also the roles of TMIE in regulating the postsynaptic nicotinic acetylcholine receptor (nAChR) function.

## 2 TRANSMEMBRANE INNER EAR

**FIGURE 2:**
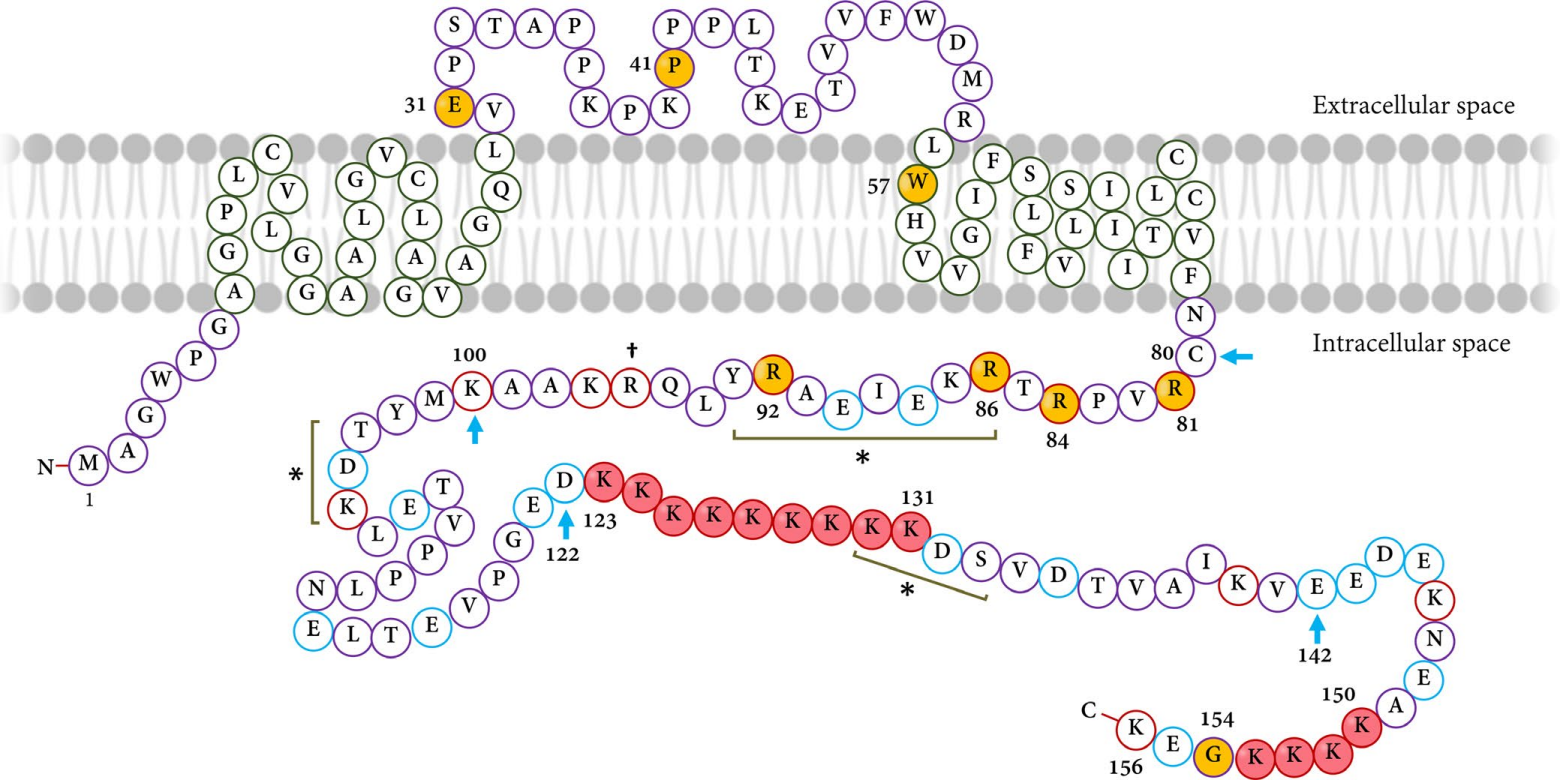
The TMIE protein structure. TMIE consists of an intracellular N‐terminus, two transmembrane parts separated by an extracellular loop and a long‐charged intracellular C‐terminus. The reported mutations are indicated by the yellow‐filled circles. The dagger (†) shows the affected residue in the counterpart of *Sr^J^* mouse. The positively charged amino acids are shown by red circles whereas negatively charged residues are indicated by blue ones. There are several lysine (K) residues in C‐terminal that are shown by red‐filled circles. Three potential protein kinase phosphorylation sites are among 86‐93, 103‐105 and 131‐133 residues—are shown by asterisks (*). The potential binding sites for the TMC1/2 proteins are among 80‐100 amino acid positions. Blue arrows highlight the regions that are the target for phosphatidylinositol 4,5‐bisphosphate (PIP2)—residues from 80 to 100 and 122 to 142. The two clusters of lysine (K) residues in the C‐terminal are indicated by red circles (from 123 to 131 and 150 to 154 residues). The figure is depicted according to the amino acid sequence and also data provided by Ref. [113]

Transmembrane inner ear gene (OMIM: 607237) is located on the 3p21 chromosomal region and consists of four exons. With a 92% sequence identity with the mouse's *Tmie*, this gene encodes a 156 amino acid protein^17^ (Figure 2). Transmembrane inner ear has an intracellular N‐terminus, two transmembrane domains separated by an extracellular loop, and an intracellular C‐terminus^21^ (Figure 2). The presence of a signal peptide is predicted by the TargetP server^22^ with a cleavage site that is located between residues 28 and 29. Homozygous or compound heterozygous mutations in the *TMIE* gene are associated with the DFNB6 that is featured by a severe‐to‐profound non‐syndromic sensorineural hearing loss along with congenital or prelingual onset (Figure 2; Table 1).

**TABLE 1:** The identified mutations in the *TMIE* gene

Nucleotide change	Amino acid change	Exon/Intron	SNP	Possible effect on gene or protein	Domain	ClinVar accession Code	References
c.92A > G	p.Glu31Gly	EX1	rs1057517839	Decreased protein stability	Extracellular	VCV000372538.2	28
c.IVS1‐2_98delAGCCCAGinsC	–	IN1	rs876657371	Splice disruption	Extracellular	VCV000003393.1	17
c.125_126insCGCC	–	EX2	rs876661301	Frameshift	Extracellular	VCV000003389	17
c.170G > A	p.Trp57Ter	EX2	rs267607120	Truncated protein	Transmembrane	VCV000003394.1	30
c.211 + 3G > C	–	IN2	rs397517865	Splice disruption	Transmembrane	VCV000047957.2	–
c.212‐2A > C	–	IN2	–	Splice disruption	Transmembrane	–	28
c.241C > T	p.Arg81Cys	EX3	rs28942096	Decreased protein stability	Intracellular carboxy‐terminus	VCV000003390.1	17
c.250C > T	p.Arg84Trp	EX3	rs28942097	Decreased protein stability	Intracellular carboxy‐terminus	VCV000003391.4	17
c.251G > T	p.Arg84Leu	EX3	rs397517866	Decreased protein stability	Intracellular carboxy‐terminus	VCV000047958.2	–
c.257G > A	p.Arg86Gln	EX3	rs750584965	Decreased protein stability	Intracellular carboxy‐terminus	–	116
c.274C > T	p.Arg92Trp	EX3	rs28941781	Decreased protein stability	Intracellular carboxy‐terminus	VCV000003392.2	17
c.460G > T	p.Gly154Ter	EX4	–	Made truncated protein	Intracellular carboxy‐terminus	–	117

Transmembrane inner ear is expressed in many human tissues, including cochlear tissues as well.^18^, ^20^ The presence of the TMIE in the spiral limbus, spiral ligament, organ of Corti and stria vascularis of the rat was also identified.^23^ Shen et al^24^ showed the expression of *Tmie* in the hair cells of the mouse organ of Corti. These studies suggest the important roles of TMIE in the hearing process. In fact, the appellation of ‘TMIE’ was due to the transmembrane domains and also its potential fundamental roles in the inner ear.^18^ In the following, we summarize some important information about TMIE and its role in the auditory system.

### 2.1 Discovery of TMIE

The discovery of the TMIE extremely benefits from the genetic studies of deafness in humans^17^ and animal models including mice^18^, ^25^ and zebrafish.^19^ In 1962, Deol and Robbins^25^ reported a new case of spinner (*sr*) mouse that manifested deafness, typical head tossing, circling and hyperactivity. Light microscopic investigation of the inner ears of the homozygote spinner mice (*sr*/*sr*) showed that the auditory and vestibular impairment was potentially peripheral in origin and no clear defects were observed in the gross inner ear morphogenesis^25^; however, an irregular apical surface of inner and outer hair cells were evident.^18^ Later studies mapped the *sr* to the distal part of chromosome 9 in mice and then comparative gene mapping investigations introduced a region of conserved synteny between the distal mouse chromosome 9 and human chromosome 3.^26^

The presence of deafness locus DFNB6 on the short arm of chromosome 3 was described for the first time in a family from southern India with second‐cousin marriage (three out of four siblings were deaf).^27^ This evidence suggested the spinner strain as a mouse model for the human non‐syndromic hearing loss caused by mutations in *DFNB6*.

Mitchem et al introduced the widening of the *Tmie* gene by a 40 kb deletion on the *sr* allele region. They reported two independent mutations in the *Tmie* gene causing HI and vestibular impairment of the spinner model.^18^ The *Tmie* gene was thoroughly deleted in the *sr* allele, whereas in *sr^J^*, a C > T substitution changed Arginine residue to a premature stop codon at a position of 96 and both cause the same phenotype (Figure 2). In sum, these studies have paved the way for the discovery of the *TMIE* gene.

### 2.2 Genotype of *TMIE*

Different mutations in the *TMIE* gene have been reported in association with severe‐to‐profound non‐syndromic hearing loss (Table 1). The first five mutations in the *TMIE* associated with DFNB6 were documented in 2002.^17^ Three of them were missense mutations in the cytoplasmic domain at the C‐terminal domain including the p.R81C, p.R84W and p.R92W, all located in the exon 3.^17^, ^27^ Two substitutions—p.R81C and p.R84W—were located at highly conserved residues, whereas the p.R92W was reported to reside in a tyrosine kinase phosphorylation region (Figure 2).^28^ The 4th mutation was a homozygous 4‐bp insertion (CGCC) in exon 2 at a nucleotide position of 125 characterized by deafness in an Indian family; this mutation was predicted to cause a frameshift mutation leading to the production of the truncated protein. The last documented mutation was a 6‐bp deletion (AGCCCA) along with a 1‐bp insertion (C) in the splice acceptor site of the intron 1 (IVS1‐2_98delAGCCCAGinsC) which was found in a Pakistani family. As an autosomal recessive mutation, it removes the acceptor splice site prior to the exon 2, therefore causing exon‐skipping that in turn results in losing some residues.^17^

The frequency of the *TMIE* mutations was reported by 1.7% among 168 Pakistani patients, whose *GJB2*‐screening was negative. Also, two new mutations were reported including p.E31G in exon 1, and c.212_2A > C at splice acceptor site of intron 2 that was predicted to result in skipping of the 3rd exon and therefore losing a part of the second transmembrane segment and half of the long C‐terminal tail of the protein.^28^ p.E31G is located in the extracellular domain in the conserved area of the TMIE.^28^, ^29^ Later, seven homozygous mutations of p.R84W and a compound heterozygous p.R84W/p.W57X were reported in the Turkish families with congenital non‐syndromic hearing loss, whose gross motor development and balance were normal; high‐resolution computed tomography (CT) examination in two patients did not show any inner ear anomalies and vestibular involvement.^30^

The frequency of p.R84W was reported by 10.3% and 2.4% in Southeastern Anatolia and Turkey, respectively.^30^, ^31^ Haplotype analysis showed that the mutation was due to a ‘Founder Effect’ since approximately 1250 years ago. Duman et al^32^ determined a frequency of 6.6% for all recognized mutations of the *TMIE* gene in 49 Turkish families with non‐syndromic hearing loss, whereas among 374 Indian families affected by autosomal recessive non‐syndromic hearing loss, the frequency of the *TMIE* gene was about 1.6%. A total frequency of 0.83% for the *TMIE* has been determined in the Iranian populations.^33^ Thus, it seems that the ‘Founder Effect’ can influence the frequency in different populations. Table 1 indicates the rest of the pathogenic or likely pathogenic *TMIE* mutations associated with HI that have been submitted to the ClinVar database,^34^ Deafness Variation Database^10^ and Human Gene Mutation Database.^35^

The C‐terminal of TMIE is rich in charged residues (41/78 amino acids) consisting of two clusters of Lysine and several Arginine residues (Figure 2). Beyond that, the C‐terminal region has three potential phosphorylation sites.^17^ A significant portion of the human gene mutations associated with the HI has been reported in such Arginine residues (Table 1), for example p.R81C, p.R84W and p.R92W impress such evolutionary conserved Arginine residues in human cause deafness, confirmed using animal models^14^, ^36^ (Figure 2). Moreover, the substitution of Arginine in the C‐terminus of TMIE, at the position of 96, has been reported as a cause for deafness in the *sr^J^* mouse, underscoring the essential role of these Arginine residues in the correct function of this protein.^18^ TMIE is proposed as a suitable candidate for connection with other functional proteins in the stereocilia; this ability is attributed to the predicted structure of the TMIE along with the special features of C‐terminal.^18^, ^28^

## 3 TMIE ANIMAL MODELS

The localization of the TMIE in the stereocilia of hair cells has been firstly indicated in the rat cochlea,^23^, ^37^ but later studies on the *Tmie^LacZ/+^* and *Tmie^LacZ/LacZ^* mice (an in‐frame insertion of a LacZ transgene into the *Tmie*) showed that the TMIE is located in the stereocilia of inner and outer hair cells next to the lower part of tip link insertion.^14^ In fact, animal models—especially the mouse and zebrafish—provide a valuable resource for studying the inherited human HI and also a helpful system for evaluating the function of the candidate genes. As a result, different proteins have been identified that are necessary for the maturation and developmental process of the human inner ear and may have a contribution with TMIE to auditory system. In the following, we summarize a snippet of information about mouse and zebrafish HI models to grasp the importance of TMIE in auditory system.

### 3.1 Mice

The *sr* mouse was the first model for human DFNB6 without auditory brainstem response.^25^ Degeneration of the hair cells, which was started at P15 and completed at P40, and the degeneration of the auditory nerve cells were also observed.^18^ These studies show that *Tmie* perturbation often causes postnatal symptoms; so due to the nature of damages, the *sr* mouse is classified into the neuroepithelial class of the mouse model.^18^ Some scrapes of evidence attribute the postnatal defects in the cochleae of *sr/sr* mice to the necessary of Tmie during the maturation of sensory cells, for example the normal development or maintenance of stereocilia bundles. Gene expression profiling of *Tmie* during the development support this notion that probably TMIE is so important in the blastocyte stage and also imperative postnatally—for example in endocrine, auditory and visual systems (Figure S1). The low number of detected *TMIE*‐associated mutations in patients can be imputed to the high risk of lethality prenatally, suggesting the critical roles of this gene in other tissues and organs.

Another mouse model for the human DFNB6 is the ‘Circling Mouse’ model (C57BL/6J‐*cir/cir*). Similar to the *sr* mouse, this model is caused by a spontaneous 40 kb deletion on chromosome 9 with complete penetrance and autosomal recessive inheritance pattern.^38^ Cho et al^38^ showed that the deletion is located between the *Lactotransferrin* (*Ltf*) and *microtubule‐associated protein 4* (*Map4*) genes on chromosome 9 that is analogous for human chromosome 3p21. Circling mice manifest profound HI in addition to hyperactive behaviour such as bi‐directional circling and head tossing. Several genes are removed in the *sr* allele embracing *K007173*, *Tsp50 Mm87012* (*Tmie*), and *mRn49018,* whereas the *cir* allele only includes the removal of the *Mm87012* (*Tmie*) and *mRn49018* genes.^18^, ^39^ *Tmie* is the only common deleted gene between the *cir/cir* and *sr/sr* mice.

Comparing the *cir/+* and *cir/cir* mice on the third day of birth (before hair cell degeneration) showed that both have similar inner ear structure, but the *cir/cir* mice did not absorb the gentamicin, gentamicin–Texas red conjugate or FM1‐43,^40^ suggesting that the maturation and mechanotransduction have impaired in the hair cells; the *Tmie* gene has been proposed to be responsible for these events.

Histological examinations in the *cir* mice indicated the postnatal degeneration of the cochlear epithelium of Corti and spiral ganglion.^39^ The neuroepithelial defects of *cir* including the irregular and shorter stereocilia bundles started earlier at 10 days and were completed more rapidly at 21 days.^20^ Collectively, TMIE has been suggested as a critical factor in the auditory system that participates in normal postnatal maturation and maintenance of stereocilia bundles of sensory hair cells.^17^, ^18^, ^39^

### 3.2 Zebrafish

The inner ear of zebrafish resembles other vertebrates—anatomically and functionally. The existence of the lateral‐line system—sense organs of the aquatic vertebrates for sensing the movements and pressure changes in the surrounding water—makes the zebrafish a useful model for peering at the molecular mechanisms in hair cells. The lateral‐line organ consists of a series of hair cell collections termed the neuromasts spread over the body surface. Expression of *tmie* is typically observed at 26, 36 and 51 hpf in zebrafish that are analogous stages of *Tmie* expression in mice.^41^

*Tmie* ^ru1000^ is a zebrafish model for human DFNB6. In this model, the *tmie* gene is mutated in two nucleotides of codon 13 from the exon 1, leading to the truncated protein with only 25 amino acids. The hair cells of this model could not successfully uptake the fluorophores that normally pass through transduction channels^42^, ^43^ and their ear structures miss the microphonic potentials in response to the vibratory stimuli.^19^ Pacentine et al^44^ showed that the ‘Gross morphology’ is normal in *tmie^ru1000^* mutant zebrafish, whereas *tmie^ru1000^* reveals subtle differences as short and abnormal hair bundles; these findings are in line with the grossly normal hair‐cell morphology observed in *Tmie* ^−/−^ mice. Owing to the defect in hair‐cell mechanosensitivity, *tmie*‐deficient zebrafish has been suggested to be somehow deaf.^44^

Studies have not been able to measure mechanotransduction currents in *Tmie*‐deficient hair cells.^14^, ^44^ Interestingly, exogenous expression of a *tmie* rescued the functional defects in *tmie* ^ru1000^ and also *Tmie*‐deficient mice.^14^, ^44^ Thus, any damage to the mechanotransduction results from the function of the TMIE protein probably generated the morphological changes in the stereocilia or hair bundles.

## 4 TMIE CONNECTS TO OTHER MEMBERS IN MECHANOTRANSDUCTION

The appropriate function of the mechanotransduction channel per se depends on some collaborations between different proteins at the tips of stereocilia.^45^ The Tip link involves two connecting proteins, the CDH23 in the upper and PCDH15 in the lower parts (Figure 1D). Both are homodimers and through their N‐terminal form a tetrameric tip link. Mutations in *PCDH15* and *CDH23* can cause the Usher syndrome and autosomal recessive non‐syndromic hearing loss.^46^, ^47^, ^48^

### 4.1 CDH23

Cadherin 23 is a member of the cadherin superfamily with multiple extracellular cadherin repeats, a single transmembrane region, and a PDZ domain‐binding interface (PBI) at its cytoplasmic C‐terminal, mediating the connection with PDZ domains. Cadherin 23 is necessary for appropriate morphogenesis of hair bundles of inner ear hair cells.^49^ Disrupted tip links and mechanotransduction have been shown in *Cdh23*‐deficiency.^50^, ^51^ Using the cytoplasmic domain of CDH23, the tip link connects to other proteins including MYO7A, harmonin (USH1C) and SANS (USH1G) that are called the ‘Upper Tip Link Density’.^52^ This connection might control the transduction by influencing the tip link proteins or by controlling hair bundle stiffness (Figure 1D).

Harmonin, MYO7A and SANS all are implicated in Usher type I (USH1) syndrome and also in different types of non‐syndromic hearing loss^53^, ^54^ (Figure 1D). Harmonin, as a scaffold protein, connects to CDH23.^55^, ^56^ Activation and adaption of the transducer channel controlled by harmonin and SANS have been offered to control the tip link assembly and mechanotransduction in which the MYO7A is essential for tensioning.^14^, ^57^ TMIE is likely to collaborate with the MYO7A in the development and maintenance of the stereociliary bundles during the postnatal developmental stages of the cochlea. The expression level of *Myo7a* increased by 3‐folds in *cir/+* and *cir/cir* against the wild‐type mice at P5, suggesting that the overexpression of *Myo7a* can be a compensatory mechanism in order to fix the damaged stereocilia in the circling mice in the absence of the TMIE protein.^58^

### 4.2 PCDH15

Protocadherin‐15 is another member of the cadherin superfamily and contains multiple extracellular cadherin repeats, a single transmembrane region, and a PBI at its cytoplasmic C‐terminal, just similar to the CDH23. The reduced tip links and altered mechanotransduction currents are typically evident in *Pcdh15*‐null or mutant mice models.^51^ Different isoforms of PCDH15—varying in their cytoplasmic domains—have been detected in the hair cells (CD1‐3).^46^ Using these cytoplasmic domains, the tip link connects to TMC1/2, LHFPL5 and TMIE that are known as the ‘Lower Tip‐Link Density’^14^ (Figure 1D, E). Beurg et al^15^ observed a Ca^2+^ entry to shorter row stereocilia upon the mechanical stimulation near the lower tip link insertion site; this study suggests the presence of a mechanotransduction channel close to the PCDH15 protein, that is by binding to the lower tip‐link density, PCDH15 converts the tip link tension into opening the mechanotransduction channel (Figure 1D, E).^59^

Protocadherin‐15 interacts with the LHFPL5 by two fragments including a transmembrane domain and a short membrane‐proximal fragment of the cytoplasmic site, which is commonly observed among different isoforms.^60^ In order to perform a ternary complex, TMIE directly connects to the PCDH15‐CD2 and indirectly attached to the LHFPL5 that in turn mediates the connection to PCDH15‐CD1 and PCDH15‐CD3.^14^ Hence, it seems that alternative splicing of the cytoplasmic domain of PCDH15 has a critical role in the specific conformation of the ternary complexes of the PCDH15, LHFPL5 and TMIE.^14^ Transmembrane inner ear not only does participate in the ternary complex but also binds to pore‐forming components of the transduction channels (Figure 1E). These can show the possible interaction between different important proteins and TMIE in the auditory system.

### 4.3 TMC proteins

Transmembrane‐Channel Like proteins (TMCs) are a conserved protein family that eight members of which (TMC1‐TMC8) have been identified in the human and mice. TMC1 mutations have been previously reported in the profound prelingual DFNB7/B11 and progressive postlingual DFNA36.^48^, ^61^ TMC1 and TMC2 proteins are located at the tip of stereocilia of hair cells,^15^, ^62^ and *Tmc1/2* KO mice show the loss of the mechanotransduction currents in hair cells.^62^, ^63^ TMC1/2 subunits form the pore of the MET channel in the hair cell^64^, ^65^ (Figure 1E).

A structural model for the TMC1 based on the ‘Transmembrane Protein 16A (TMEM16A)’ indicated the existence of a large cavity near the protein‐lipid, proposing that it could function as a permeation pathway.^65^ TMEM16A bears sequence similarity to the TMCs and belongs to a family of membrane proteins containing eight transmembrane segments; its expression is associated with calcium‐activated chloride channel activity (reviewed in Ref. [66]). Moreover, truncated turtle TMC1/2 proteins embraced with the artificial lipid bilayers have been indicated to form the mechanically activated ion channels.^67^ Intracellular C‐terminus of PCDH15 has been indicated to interact with the N‐terminus of the TMC1,^68^, ^69^ and there is a possible association between a dimer of PCDH15 and a dimer of TMC1/2. In this complex, each monomer of PCDH15 may bind to each TMC monomer to control the opening of the pore.^64^

There is a probably direct association between TMIE and TMC1/2, specifically in *tmie* mutant zebrafish in which the Tmc proteins could not deal with targeting hair bundles, whereas *tmie* overexpression promotes bundle localization of Tmc proteins; thus, it seems fair to conclude that the second transmembrane domain and adjacent regions of Tmie are of importance for proper targeting Tmcs into hair bundles.^44^

### 4.4 LHFPL5

LHFP‐like protein 5 is an essential component for conductance and adaptation characteristics of the transducer channel.^60^ Structurally and functionally, this protein has a similarity with the ‘Transmembrane AMPAR Regulatory Proteins’ that allosterically control the pore properties of glutamate receptors.^60^ Mutations in *LHFPL5* are associated with human DFNB67^70^ and have been also reported to cause vestibular dysfunction and deafness in the mice as a result of severe degeneration of Corti.^71^ During the otocyte development, LHFPL5 is located throughout the bundle, but with the onset of hearing or postnatally, it gradually moves to the tips of the shorter stereocilia. LHFPL5 cannot be localized at the tip of stereocilia in the *Pcdh15*‐deficient mice^72^ and also is essential for localization of the PCDH15 to the site of mechanoelectrical transduction.^60^, ^73^ In the absence of the LHFPL5 in the cochlear hair cells, the current of mechanoelectrical transduction is not completely suppressed,^60^ whereas the number of tip links significantly decreased.^60^ LHFPL5 is also involved in the correct localization of the TMC1 in the mouse cochlear hair cells,^69^ although new evidence demonstrates that the Tmc1 and Tmc2b proteins can localize in an independent of the Lhfpl5 in the stereocilia of the zebrafish hair cells.^74^

### 4.5 CIB2

Calcium and integrin‐binding protein 2 is involved in the normal operation of mechanotransduction machinery in the auditory hair cells^75^ (Figure 1E). Although it is not a critical factor for localization of the PCDH15 or TMC1/2, this protein connects to the N‐terminal domain of the TMC1.^76^ CIB2 contributes to the intracellular Ca^2+^ signalling,^77^ which is called for the hearing process (reviewed in Ref. [78]). The *CIB2* mutations—associated with DFNB48—change the CIB2 connectivity with the TMC1/2.^76^ The mechanoelectrical transduction currents were abolished in the auditory hair cells of the *Cib2*‐deficient mice^75^, ^76^ and morphological abnormalities of the hair cells started only after birth‐time that led to the regression of the stereocilia and rapid death of the hair cells.^75^, ^79^ Recently, Tang et al, using *C* *elegans* models, showed that TMC1 connects to the cytoskeleton through CIB and ankyrin proteins. They proposed the probability of transmitting force to the channel through ankyrin^80^ (Figure 1E).

## 5 TMIE IN MECHANOTRANSDUCTION

Tmie shows a normal trafficking pattern to the top of stereocilia even in the absence of the individual mechanotransduction proteins including the Pcdh15a, Lhfpl5a or Tmc1/2.^44^ This protein is called for correct localization of the Tmc1 and Tmc2b in the hair bundle, that is overexpression of the *tmie* can increase the localization of the Tmc1/2b to the hair bundle.^44^ In *Tmie* ^−/−^ mice, Cunningham et al observed no TMC1 in the stereocilia, whereas the expression of *Tmc2* was significantly decreased. On the other hand, the localization of other mechanotransduction components including the PCDH15, CDH23 and LHFPL5 in the stereocilia had not been changed in the *Tmie* ^−/−^ mice^36^. Although TMIE was localized correctly to the stereocilia in the absence of other mechanotransduction components, mechanotransduction currents could not be evoked. This study proposed that there is a undeniable connection between TMC1/2 proteins and TMIE, that is without any contribution of TMIE, TMC1/2 cannot perform a functional mechanotransduction channel in the hair cells.^36^, ^44^

Recently, the role of the different parts of TMIE in the mechanosensitivity of the hair cells has been investigated.^36^, ^44^ Transmembrane inner ear connects to TMC1/2 through its C‐terminal domain that is located near its plasma membrane.^36^ Moreover, TMIE binds to the phosphatidylinositol 4,5‐bisphosphate (PIP2) from two parts of the C‐terminal cytoplasmic domain (residues of 80‐100 and 122‐142) (Figure 2).^36^ TMIE’s binding to the PIP2 is disrupted in the presence of mutated Arginine residues at positions of 82, 85 and 93 associated with the hearing loss.^36^ The expression analysis of the protein with these three point mutations in mechanosensory hair cells showed that p.R93W mutation affects the TMIE localization on the stereocilia.^14^, ^36^ The p.R82C and p.R85W had a similar pattern of membrane localization (similar to the wild‐type mice) but caused mechanotransduction currents to drastically be reduced.^14^ The p.R82C and p.R85W mutations influence the localization of the TMC1 and, to some extent, TMC2 in the hair bundles.^14^, ^36^ In the mouse hair cells with p.R82C mutation, the mechanotransduction channel is sensitized to the PIP2 depletion than the wild‐type mice. The model showed a faster decline in transducing currents, probably because of the decreased coupling of the TMIE to the PIP2 in the plasma membrane. p.R82C is located in a domain that is essential for TMC1/2 binding; therefore, it can be concluded that part of its effect on transduction will be due to the disrupted interactions among the PIP2, TMIE, and TMC1/2.

Structural modelling has recommended that the TMC1 has a large cavity next to the protein‐lipid interface.^65^ Cunningham et al^36^ proposed that the second transmembrane of the TMIE couples with this pore region and protects the pore from the lipid environment; indeed, it has been identified that the PIP2 binding domain of TMIE is next to the pore region; hence, there is a probability that influences the pore properties. The R82 is located immediately near the second transmembrane domain of the TMIE (Figure 2); hence, the mutation might influence this domain. The binding of a similar structure with the pore can influence the conductance properties of the channels in the ‘mechanosensitive channel of small conductance’ and ‘Volume‐Regulated Anion Channels’.^81^, ^82^ In addition, a similar charged cytoplasmic domain has been reported in the mechanically gated ‘TWIK‐related K^+^ channel 1 (TREK‐1)’ channel. This domain connects to PIP2 and manages the coupling of TREK‐1 to the membrane and channel gating.^83^, ^84^

There is a kind of controversy about the N‐terminal of the TMIE. Pacentine et al^44^ declared the first putative helix is dispensable, whereas another study showed that the deletion of the N‐terminus (containing the first transmembrane domain) influences the channel gating and transmission.^36^ On the other hand, Cunningham et al showed that the deletion of the N‐terminal part of the TMIE that includes the first transmembrane domain affects the channel gating and force transmission that may be explained by the perturbations in the connection of the TMIE with other components of the mechanotransduction complex such as the LHFPL5 and PCDH15 connecting to the TMIE.^14^, ^36^

## 6 MECHANOTRANSDUCTION AND MORPHOGENESIS

The number of mechanotransduction channels is increased rapidly in cochlear hair cells around the time of birth in mice and rats.^85^, ^86^ At all developmental stages, it has been identified that the morphological maturation of the hair bundles is not fully complete, that is mechanotransduction currents are necessary to fulfil the morphogenesis of such hair bundles (reviewed in Ref. [87]). Indeed, a threshold level of transduction current activity may determine whether a stereocilium should either incorporate into the bundle or be resorbed as a microvillus.^87^ It seems that calcium entering the cell through MET channels can destabilize calcium‐sensitive crosslinks between actin filaments in the microvilli.^87^

Generally, inner hair cells of the rodent cochlea have three rows of stereocilia: whereas the first row is tall and thick, the second one is short and thick; besides, the third one is short and thin. A collection of five proteins including the MYO15A‐S (the short isoform of MYO15A), epidermal growth factor receptor pathway substrate 8 (EPS8; actin regulator), Whirlin (WHRN; scaffolding protein) and G‐protein‐signalling modulator 2 (GPSM2), and guanine nucleotide‐binding protein (G protein) alpha inhibiting 3 (GNAI3) polarity proteins are known as the marker protein for row 1 that is involved in elongation of the actin core of the stereocilia. Likewise, MYO15A‐L (the long isoform of MYO15A), the heterodimeric capping protein subunit CAPZB and its partner TWF2, and EPS8L2 are located close to each other at the tip of row 2.^86^, ^88^, ^89^ Recently, Krey et al have reported an alteration in the stereocilia lengths and diameters in the *Tmie* or *Tmc* KO mice; the given phenotype in *Tmie* KO was severe than *Tmc* KO mice. These mice show uniform row 1‐3 diameters and a smaller distinction in length between rows. Besides, the distribution of marker proteins has also changed for each row^86^. In the hair cells of *Tmc1^KO/KO^*; *Tmc2^KO/KO^* and *Tmie^KO/KO^* mice, the distribution of the EPS8, MYO15A, GNAI3, CAPZB and TWF2 is changed. Widening of the cochlear stereocilia is correlated with the acquisition of mechanotransduction and the transduction channels specify and maintain the row identity and control the stereocilia's length and diameter.^86^ As discussed, the genetic investigation of HI has revealed various required genes for hair‐bundle morphogenesis, among these, human families with deafness can be used fruitfully as a resource to detect genes that are required for hearing and hair‐bundle morphogenesis. We believe that future investigations can unveil the molecular mechanisms behind the hair‐bundle morphogenesis and maybe through accompanying TMIE.

## 7 TMIE AND PIP2 SIGNALLING PATHWAY

Stimuli sensed by hair cells increase tension in the tip links that in turn convert the physical forces into chemical signals using inner cells’ Ca^2+^ signalling pathways. The increased amount of stereociliary Ca^2+^ levels trigger MET channel closure through adaptation—a negative‐feedback mechanism containing of a shift of the sensitive range of the MET process.^90^ Ca^2+^ may also affect the function of MET channels indirectly—for example via adenosine 3ʹ,5ʹ‐cyclic monophosphate (cAMP)—because the rise of stereocilia Ca^2+^ concentration can promote the Ca^2+^‐calmodulin‐activated type I adenyl cyclase of the hair cells that in turn is followed by activation of protein kinase A and phosphorylation of relevant targets. The increase in the stereocilia Ca^2+^ concentration weakens calmodulin binding to the myosin 1c IQ motifs, which in turn interacts with anionic phospholipids in the membrane such as PIP2.^91^, ^92^

**FIGURE 3:**
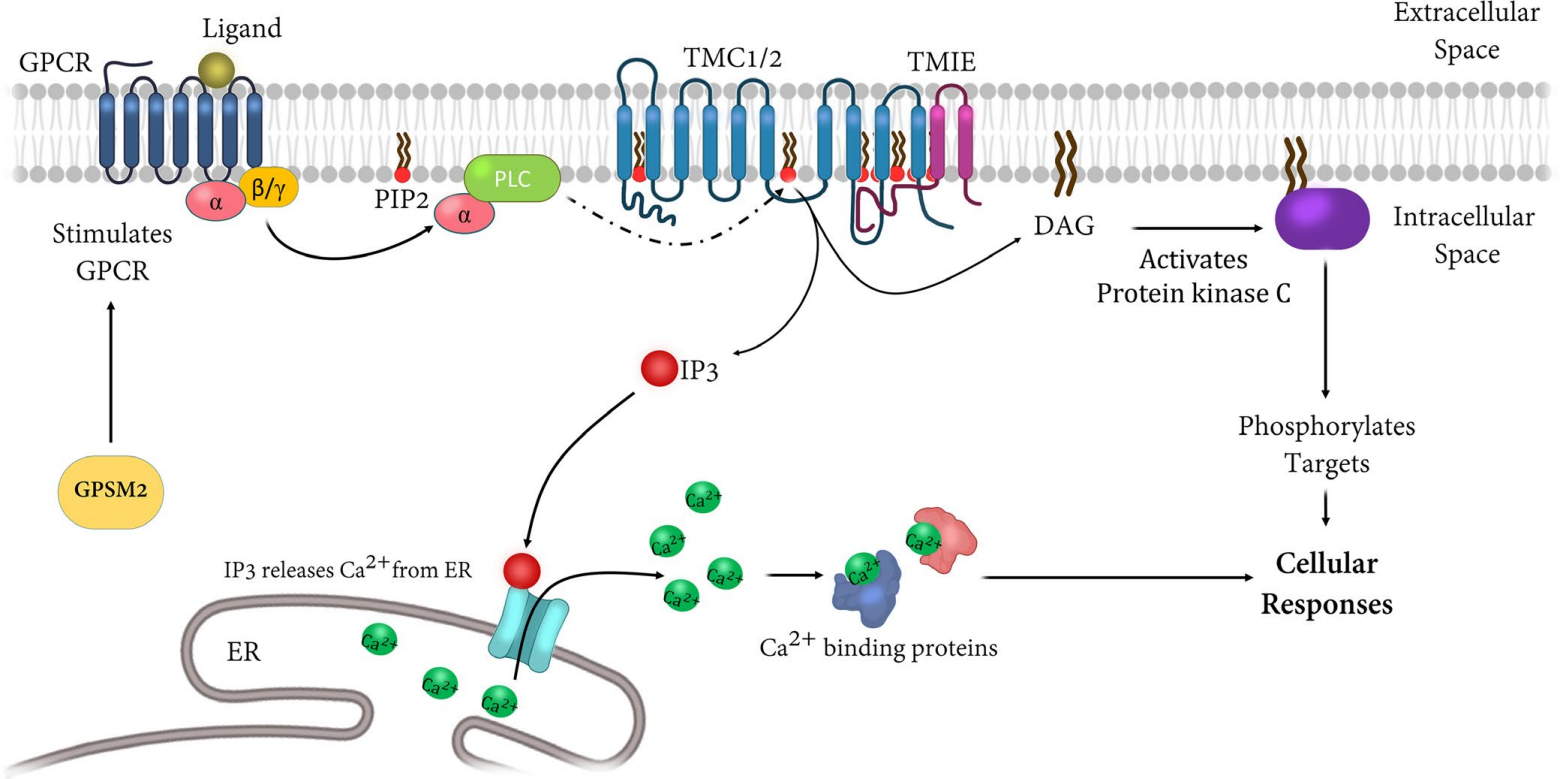
Schematic pathway of PIP2 and its possible contribution to the auditory system. Stimulation of receptors coupled to Gα activates Phospholipase C (PLC), which leads to the release of diacylglycerol (DAG) and IP3. GPSM2 is a regulator of G protein‐coupled receptor signalling that, in turn, stimulates phospholipase C (PLC). The C‐terminal cytoplasmic TMIE domain contains charged amino acids that mediate binding to phospholipids, including PIP2. DAG remains membrane‐associated and activates protein kinase C, whereas IP3 diffused into the cell and stimulates the IP3 receptor in the endoplasmic reticulum (ER), leading to mobilization of intracellular Ca^2+^ stores. PKC phosphorylates the targets and therefore induces cellular responses including. The figure is redrawn from Refs [59, 114]

Phosphatidylinositol 4,5‐bisphosphate is a prominent component of the plasma membrane that can alter the function of ion channels.^93^ GPSM2 is a regulator of G protein‐coupled receptor signalling that, in turn, stimulates phospholipase C (PLC) and is required for normal hearing. GPSM2 along with its partner—GNAI3—is expressed asymmetrically at the apical surface of hair cells, and control the localization of kinocilia.^86^, ^94^ PLC‐dependent hydrolysis of PIP2 generates the second messenger molecules: diacylglycerol and inositol trisphosphate (IP3); the latter binds to IP3‐receptors to activate Ca^2+^ efflux from the endoplasmic reticulum, raising the cytosolic free Ca^2+^ concentration. PIP2 is often scattered on the membrane of the tip of stereocilia next to the mechanotransduction channel in the auditory sensory hair cells of the rats. It may adjust the channel configuration to change the calcium permeation and single‐channel conductance (Figure 3). Modulating the amount of free PIP2 in the inner hair cell stereocilia without altering the hair bundle compliance can change the channel resting open probability, ion selectivity, conductance, adaptation and amount of calcium.^95^, ^96^ The cavity formed by the protein conformation at the protein‐lipid interface in the TMC structural model based on the TMEM16 proteins suggested the possible role of the membrane lipids such as the PIP2 in regulating the activity of the mechanotransduction channel.^65^

It has been shown that the C‐terminal cytoplasmic TMIE domain involves some positively charged residues that mediate binding to phospholipids, especially PIP2,^36^ and also C‐terminal TMIE affects its binding to TMC1/2, indicating that some of the previously known PIP2 effects on channel function may be mediated by TMIE. This may result in alternations in channel conductance and ion selectivity, suggesting that this part of TMIE regulates the pore properties of the transducer channel. Interestingly, the depletion of PIP2 from hair cells affects MET; this effect is stronger in p.R82C mutant in comparison with the wild‐type models.^97^ It still is unclear whether the PIP2 dependence arises from its direct interaction with the channel complex or from an indirect effect on lipid mechanics.

## 8 TMIE IS A SUBUNIT FOR α9α10 nAChR

Nicotinic Acetyl‐Choline Receptor (nAChR) is a nicotinic family of cholinergic receptors and α9α10 nAChR counts as a member of this family that is situated in cochlear and vestibular hair cells.^98^ The α9α10 nAChR participates in synaptic currents that originated from medial olivocochlear neurons. The α9α10 nAChR is among the most calcium selective ligand‐gated channels which connect to the calcium‐activated SK2 potassium channel in the base of hair cells.^98^

**FIGURE 4:**
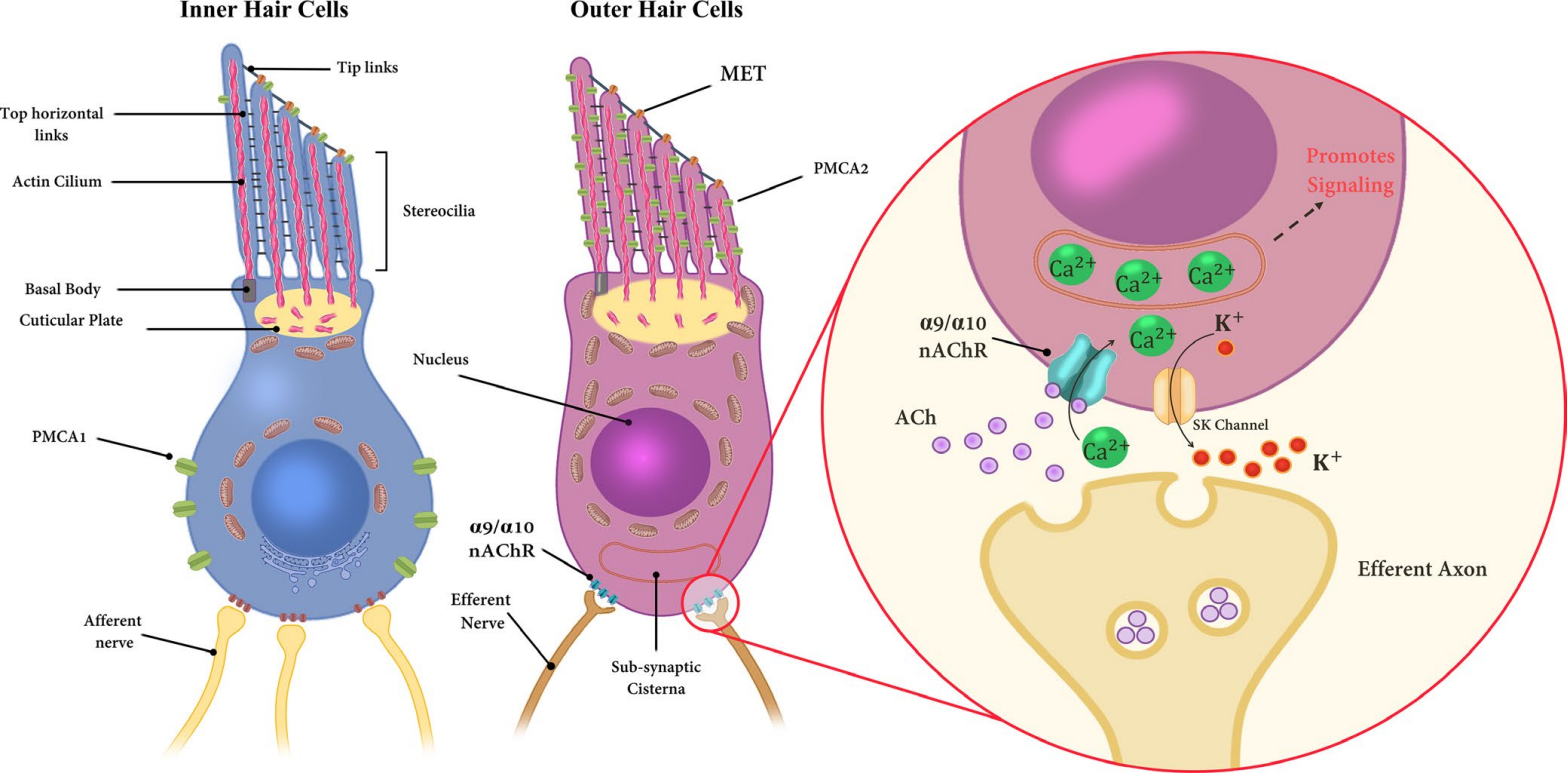
Detailed diagram of the synaptic terminal of afferent and efferent neurons onto outer and inner hair cells. Ca^2+^ enters via mechanotransducer (MET) channels in the stereocilia of both hair cell types, and also via voltage‐dependent Ca^2+^ channels (mainly in inner hair cells) and nicotinic acetylcholine receptors (nAChR) in outer hair cells. The efferent nerve's terminals release acetylcholine (ACh), which activates α9α10 nicotinic receptors (nAChRs) in outer hair cells. Ca^2+^ influx through these receptors activates SK or potassium (K^+^) channels (is depicted as a yellow channel). A Ca^2+^ store is always observed in direct opposition to the location of the nicotinic receptors. Ca^2+^ can trigger the signalling pathways in the target cell (Hair cells). Two types of Ca^2+^ ATPase pumps exist in hair cells extruding the Ca^2+^ including PMCA1 in inner hair cells and PMCA2 in outer hair cells. The figure is redrawn from Ref. [115]

New evidence determined that hair cell α9α10 nAChR functional expression is regulated by ligand binding and the coexpression of either TMIE and TMEM132e.^99^ *TMEM132E* is deafness‐associated gene.^100^ This study introduces TMIE as the α9α10 auxiliary subunit. Moreover, it has been identified that aberrant up‐regulation of neonatal α9α10 channel function as well as abnormal persistence of cholinergic innervation and α9α10 synaptic transmission beyond P12 in *Tmie* mutant mice.^99^ In agreement with these results, the presence of TMIE in the cell body of hair cells has been demonstrated previously^36^ and mRNA expression of *TMIE* is also enriched together with SK2, α9 and α10 in outer hair cells.^101^, ^102^ All in all, these data confirm that TMIE is an auxiliary subunit that participates in channel gating of α9α10 nAChR (Figure 4) and provides a mechanism to couple cholinergic innervation to postsynaptic nAChR expression and probably enables drug discovery for auditory disorders associated with these hair cell receptors.

## 9 THE THERAPEUTIC PERSPECTIVE OF TMIE

The medial olivocochlear bundle decreases the gain of the cochlear amplifier through reflexive activation by sound. This system improves sound discrimination, refines tonotopic mapping, and protects against sound‐induced HI.^98^, ^99^ These specifications are a suitable pharmacological target for acoustic trauma, presbycusis, and tinnitus.

Moreover, the pharmacological potential of α9α10 nAChR has been always noticed.^103^, ^104^, ^105^ Some studies showed that when cholinergic activity through α9α10 nAChR is enhanced, it could lead to the protection and even repair of the inner ear sensory epithelium from acoustic trauma damages.^104^, ^105^ Furthermore, increasing efferent innervation of inner hair cells was observed in age‐related mouse models.^106^ Generally, this emerging evidence—introducing deafness‐associated *TMIE* gene as an encoding subunit of α9α10 nAChR in the medial olivocochlear system—increases a new therapeutic perspective for auditory and vestibular disorders.

Hair cell morphology emerges normally in the *Tmie*‐deficient mice at early postnatal ages, which might provide a therapeutic opportunity for the treatment of TMIE‐related sensorineural deafness. Notably, several other mechanotransduction components such as TMC1, USH1C, LHFPL5, CDH23, and MYO15A have been applied in gene manipulation/therapy for the treatment of the HI (reviewed in Refs [107, 108]). The essential role of the TMIE in mechanotransduction in addition to its close collaboration with other proteins makes it a good candidate for prevention, preservation, and repairing‐based therapies for HI.

According to Delmaghani et al,^109^ there are plenty of feasible approaches that can be used for inner ear gene therapies to touch upon viral and non‐viral vectors, gene replacement, gene suppression—RNA‐based therapies, CRISPR/Cas9‐based genome editing, auditory hair cell regeneration, and protective local treatments. The most common form of gene therapy involves the delivery of a functional or therapeutic ‘transgene’ to the target cells to replace or complement the defective gene responsible for the disease in general. Different clinical trials of gene replacement therapy for inner ear and central hearing disorders—caused by biallelic recessive and loss‐of‐function dominant mutations—have broadened the horizons towards using such a strategy in the treatment of auditory and/or vestibular conditions; for instance, several gene therapy trials including those for the autosomal recessive gene *MYO7A* causing Usher syndrome are being undertaken.^110^

Additionally, other investigations on different models of deafness have confirmed the efficacy of gene supplementation for amending various inner ear defects, for example a total restoration of vestibular function and somehow a less complete restoration of hearing were observed in models with defects in harmonin, sans, and whirlin proteins (reviewed in Ref. [109]). Various approaches have been performed to restore the proper functions to the MET channels as well, for example intracochlear neonatal AAV‐mediated SaCas9‐KKH‐gRNA delivery has been demonstrated that prevent deafness in *Tmc1* ^Bth/WT^ mice for up to one‐year post‐injection, raising hopes to treat this gene‐related deafness in patients.^111^ Interestingly, in another mouse model for DFNB7/11 recessive deafness with a defect in *Tmc1*, round window membrane injections of synthetic AAV2/Anc80L65 encoding Tmc1 resulted in the approximately complete restoration of auditory and vestibular function and morphological rescue.^112^ Although there are no clinical trials for TMIE in human or animal models, due to the critical roles of this protein in the MET channel, it seems to be viable in a not‐so‐distant future.

## 10 CONCLUSION AND FUTURE PERSPECTIVES

The cochlea and the organ of Corti are fascinating structures in which the mechanoelectrotransduction transpires. Over the last 20 years, remarkable steps have been taken forward to a better understanding of this process. Although it has achieved high goals, some obscure points must be unravelled; for example, many genes have been identified that contribute to the hearing process through encoding intracellular motors, adhesion proteins or even scaffolding proteins in the inner ear. However, it is axiomatic that there are still other genes and pathways that remain to be detected. Nonetheless, with the advent of additional high‐tech molecular and genetic tools, for example single‐cell transcriptomics, it appears likely that the pace of discovery will increase.

Pioneering genetic and physiological studies have indicated a pivotal role of the TMIE protein in the hearing process. Transmembrane inner ear is a critical component of the mechanotransduction machinery of the hair cells and directly and indirectly contributes to the functional molecular mechanism of maturation, development and maintenance of the hair cells; however, in total little is known about its contribution to the hearing process. In this review, we summarized some important findings to illustrate the molecular mechanisms whereby TMIE plays role in the hearing process. We believe that future studies can remove the veil of ignorance and answer the some obscure aspects, for example it is unclear how epigenetic modulations (eg methylation and acetylation) or environmental interventions affect the hearing process through TMIE or MET complex. We still do not know much about how TMIE contributes to controlling α9/α10 nAChR and also plays roles in the MET complex; or is there any relationship between these two different functions? The viable use of TMIE as a target for gene therapy/replacement yet still remains blanketed in mystery. We believe that foreseeable investigations will shed light on TMIE protein and its contribution to the hearing process. This can in turn provide valuable information about the biological aspects of ‘hearing’, which will pave the way to utilize it effectively for therapeutic purposes.

## CONFLICT OF INTEREST

The authors have no conflict of interest to declare.

## AUTHOR CONTRIBUTION

**Mohammad Farhadi:** Writing‐original draft (equal). **Ehsan Razmara:** Visualization (equal); Writing‐original draft (equal); Writing‐review & editing (equal). **Maryam Balali:** Writing‐original draft (equal). **Yeganeh Hajabbas Farshchi:** Visualization (lead). **Masoumeh Falah:** Conceptualization (equal); Writing‐original draft (equal); Writing‐review & editing (equal).

## Supporting information

Figure S1

## Data Availability

The paper is exempt from Data sharing.
