# Supplementary figures and images for: How Transmembrane Inner Ear (TMIE) plays role in the auditory system: A mystery to us
Source: J Cell Mol Med. 2021 May 13;25(13):5869–83. doi: 10.1111/jcmm.16610 (PMC8256367; doi:10.1111/jcmm.16610)

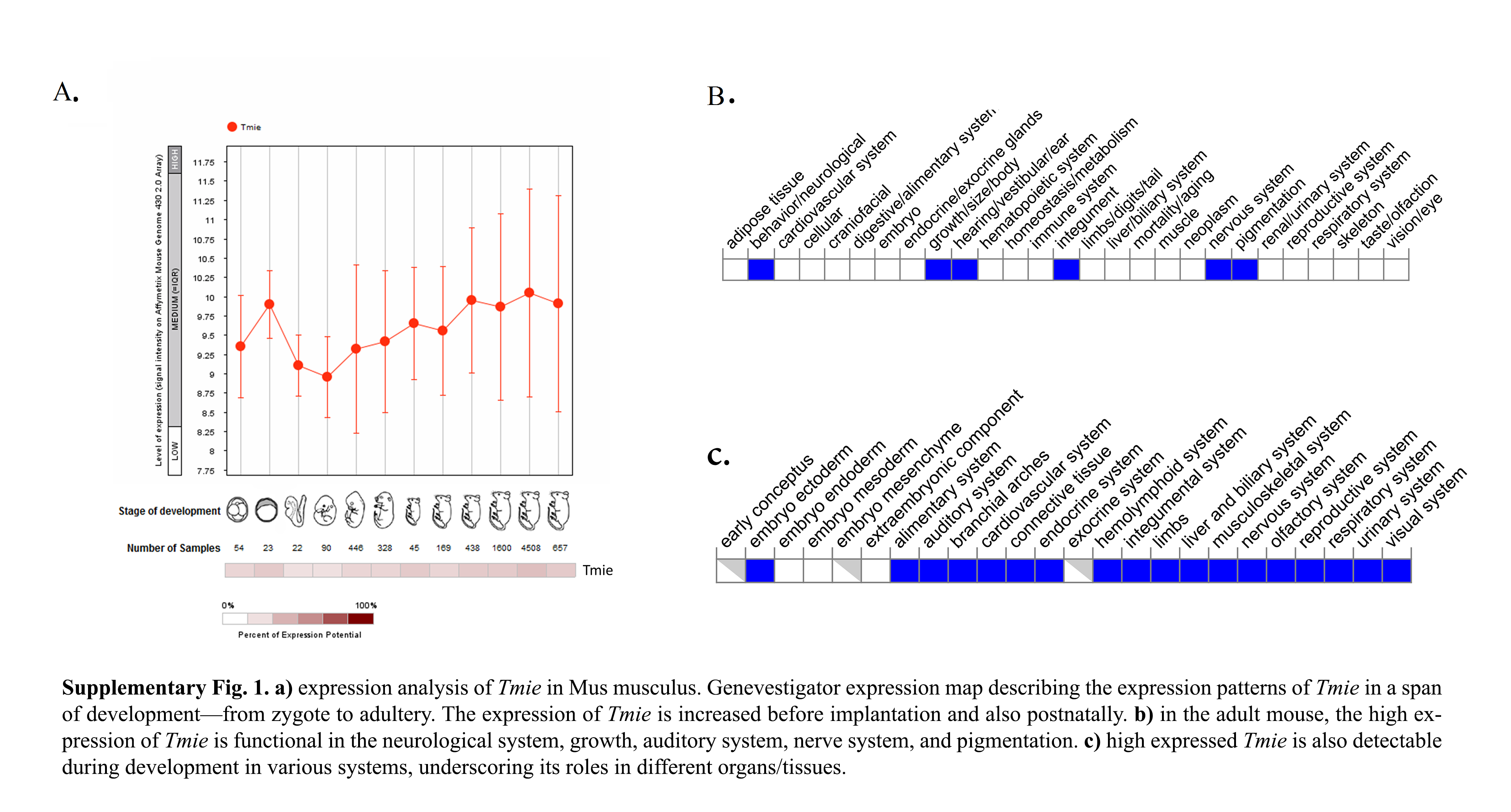

Supplement: Supplementary file 1 — Figure S1 [file JCMM-25-5869-s001.tif]
